# Supplementary material for: Classroom Movement Breaks Reduce Sedentary Behavior and Increase Concentration, Alertness and Enjoyment during University Classes: A Mixed-Methods Feasibility Study
Source: Int J Environ Res Public Health. 2021 May 24;18(11):5589. doi: 10.3390/ijerph18115589 (PMC8197210; doi:10.3390/ijerph18115589)
Supplement: Supplementary file 1 [file ijerph-18-05589-s001.zip › ijerph-1211190-supplementary.pdf]

Participant questionnaire

Student number: ________________

Ethics reference number HEC19527

Demographic details

What is your gender? ☐ Female ☐ Male ☐ Non-binary; fluid; queer ☐Prefer not to answer

What is your current age? _______years

Are you enrolled as an international student? ☐ Yes ☐ No

What physiotherapy course are you enrolled into?

☐ Graduate Entry Masters Program

☐ Bachelor of Applied Sciences and Master of Physiotherapy Program

Are you a Bendigo or Bundoora student?

☐ Bendigo

☐ Bundoora

Week 2

Class 1

Please indicate your overall perceived level of alertness, concentration and enjoyment throughout the class by drawing a line at the appropriate point on each line below.

Class: EBP / CDM (please circle) **Movement breaks: Yes/No (please circle)**

Overall level of mental alertness (your ability to think quickly and notice things)

0 ______________________________ 10

Least alert Most alert

Overall level of concentration (your ability to focus your attention without getting distracted)

0 ______________________________ 10

Least concentration Most concentration

Overall level of enjoyment

0 ______________________________ 10

Least enjoyable Most enjoyable

Class 2

Class: EBP / CDM (please circle) **Movement breaks: Yes/No (please circle)**

Overall level of mental alertness (your ability to think quickly and notice things)

0 ______________________________ 10

Least alert Most alert

Overall level of concentration (your ability to focus your attention without getting distracted)

0 ______________________________ 10

Least concentration Most concentration

Overall level of enjoyment

0 ______________________________ 10

Least enjoyable Most enjoyable

LA TROBE UNIVERSITY

Week 3

Class 1

Please indicate your overall perceived level of alertness, concentration and enjoyment throughout the class by drawing a line at the appropriate point on each line below.

Class: EBP / CDM (please circle) **Movement breaks: Yes/No (please circle)**

Overall level of mental alertness (your ability to think quickly and notice things)

0 ______________________________ 10

Least alert Most alert

Overall level of concentration (your ability to focus your attention without getting distracted)

0 ______________________________ 10

Least concentration Most concentration

Overall level of enjoyment

0 ______________________________ 10

Least enjoyable Most enjoyable

Class 2

Class: EBP / CDM (please circle) **Movement breaks: Yes/No (please circle)**

Overall level of mental alertness (your ability to think quickly and notice things)

0 ______________________________ 10

Least alert Most alert

Overall level of concentration (your ability to focus your attention without getting distracted)

0 ______________________________ 10

Least concentration Most concentration

Overall level of enjoyment

0 ______________________________ 10

Least enjoyable Most enjoyable

Week 4

Class 1

Please indicate your overall perceived level of alertness, concentration and enjoyment throughout the class by drawing a line at the appropriate point on each line below.

Class: EBP / CDM (please circle) **Movement breaks: Yes/No (please circle)**

Overall level of mental alertness (your ability to think quickly and notice things)

0 ____ 10

Least alert Most alert

Overall level of concentration (your ability to focus your attention without getting distracted)

0 ____ 10

Least concentration Most concentration

Overall level of enjoyment

0 ____ 10

Least enjoyable Most enjoyable

Class 2

Class: EBP / CDM (please circle) **Movement breaks: Yes/No (please circle)**

Overall level of mental alertness (your ability to think quickly and notice things)

0 ____ 10

Least alert Most alert

Overall level of concentration (your ability to focus your attention without getting distracted)

0 ____ 10

Least concentration Most concentration

Overall level of enjoyment

0 ____ 10

Least enjoyable Most enjoyable
